# Discussions by Elders and Adult Children About End-of-Life Preparation and Preferences

**Published:** 2007-12-15

**Authors:** Anne P Glass, Lusine Nahapetyan

**Affiliations:** Institute of Gerontology, College of Public Health, University of Georgia Institute of Gerontology; Health Policy and Management, College of Public Health, The University of Georgia, Athens, Georgia

## Abstract

**Introduction:**

In the United States, 73% of deaths occur among people aged 65 years or older. Although most would prefer to die at home after a short illness, most actually die in institutions after prolonged declines. Despite this discrepancy, elders and their adult children often do not discuss end-of-life preferences. Use of advance directives has not been widespread, and people often avoid the subject until a crisis. This project focused on informal family communication about end-of-life preparation and preferences, about which little is known.

**Methods:**

In May 2006, we conducted in-depth exploratory interviews with 15 older adults about their end-of-life preparation and preferences and with 15 younger adults about their parents' end-of-life preparation and preferences. The interview included an item rating the depth of discussion.

**Results:**

Participants in both groups were primarily female and white. Mean age of older adults was 78.6 years (range, 70–88 years). Mean age of younger adults was 53.1 years (range, 42–63 years); mean age of their parents was 82.6 years (range, 68–99 years). Nine older adults reported discussing end-of-life preparation and preferences with their adult children; six had barely discussed the topic at all. Ten younger adults reported having talked with their parents about end-of-life preparation and preferences; five had not discussed it. Barriers to discussions about end-of-life preparation and preferences were fear of death, trust in others to make decisions, family dynamics, and uncertainty about preferences. Facilitators for discussion were acceptance of the reality of death, prior experience with death, religion or spirituality, and a desire to help the family. Successful strategies included casually approaching the topic and writing down end-of-life preparation and preferences.

**Conclusion:**

Knowing the obstacles to and facilitators for discussion can help health care and public health professionals target approaches to encouraging elders and their families to discuss end-of-life preparation and preferences before a crisis.

## Introduction

In the United States, death is increasingly the province of old age, with 73% of deaths occurring among people aged 65 years or older (1). End-of-life care is mediocre at best (2) and therefore is an emerging health concern (3,4). Most people express a desire to die at home after a short illness, but 75% will die in institutions — half in hospitals and 25% in nursing homes — after slow declines caused by chronic disease (2). Twenty-five percent of Medicare expenditures for an average beneficiary occur in the final year of life (5).

People can increase the likelihood that end-of-life care will meet their wishes by communicating in advance those wishes to others. Advance directives (i.e., living will and health care power of attorney) have been advocated since at least 1990 when Congress passed the Patient Self-Determination Act, but they still are not widely used (6,7). Because little is known about the process of informal family discussions regarding end-of-life preparation and preferences (EOLPP), we studied the perspectives of 15 elders about their EOLPP and 15 younger adults about their parents' EOLPP. We sought to answer the following questions: 1) How do elders express their EOLPP to their children? 2) Are their children receptive? 3) What are the barriers to this exchange of information? 4) What facilitates these discussions? and 5) What differences emerge from examining the older and younger adults' responses separately?

## Methods

### Descriptive information

Because death remains a taboo subject in modern U.S. society, we chose a qualitative design based on constructivist perspectives (8) for this exploratory pilot project. When little is known about a subject, qualitative research is appropriate to harvest personal perceptions regarding the topic.

In May 2006, after obtaining approval from the University of Georgia Institutional Review Board, we conducted in-depth interviews with 15 community-dwelling persons aged 70 years or older (i.e., older adults [OAs]) who had living children and with 15 persons aged 42 to 63 years (i.e., younger adults [YAs]) who had parents living independently. We identified participants through purposive sampling, using the snowball technique. Seven OAs were recruited through the local council on aging, and four were recruited through acquaintances who then suggested four others, consistent with the snowball approach. YAs were similarly recruited: four were staff or volunteers at the local council on aging; eight were recruited through personal acquaintances; and three were recruited through snowballing. We did not attempt to pair parents with their own adult children but instead chose OAs and YAs independently. Participants received a $25 honorarium.

### Interview questions

Our overall goal was to develop and pilot a guide for comprehensive qualitative in-depth interviews for a larger study related to death and dying. The first author conducted all interviews. Using open-ended questions, we inquired about participants' experiences with the deaths of family members and friends; knowledge about and use of hospice; and attitudes and feelings about death, funerals, and related topics. Next we asked OAs about the process and quality of discussions with their adult children about EOLPP and sought the same information from YAs regarding conversations with their parents. Because the interviews queried attitudes about both funerals and end-of-life care, responses varied in addressing one or both topics. We asked participants about their familiarity with *Five Wishes* (9), which incorporates the living will and health care proxy in an easy-to-understand format that is useful for family discussions.

We solicited demographic information about participant age, sex, race/ethnicity, and education; OAs' number and ages of adult children; and the ages of the YAs' and their parents, as well as YAs' number of siblings. Two ratings questions asked OAs to self-report their health and YAs to report their parents' health on a scale of 1 (poor) to 5 (excellent); and participants to rate the depth of discussions about EOLPP with their children (OAs) or parents (YAs) from 1 ("hardly discussed at all") to 7 ("have discussed completely and taken action"). Interviews averaged 60 to 90 minutes.

### Analysis

We used several methods for addressing rigor in qualitative research (8,10-12). We kept meticulous records of interviews, which were audio taped and transcribed verbatim. We reviewed the transcripts while listening to the interview tapes to ensure accuracy. Transcripts were entered into the NVivo 7 qualitative analysis software (QSR International, Cambridge, Massachusetts), which was used for coding themes. Two researchers from different disciplines independently coded the transcripts through multiple iterations of coding and constant comparison; an audit trail was maintained documenting how the themes emerged.

## Results

OAs were primarily female (13 [87%]) and white (10 [67%]), with four (27%) African Americans and one (7%) Asian. Mean age of OAs was 78.6 years (range, 70–88 years). OAs had a mean of 3.2 adult children ranging in age from 36 to 64 years (mean: 50.1 years). Four OAs had some high school; three were high school graduates, five had at least some college, and two had attended graduate school; for one OA, education was unknown. OAs' self-rated health averaged 3.50; none reported their health as poor.

YAs also were primarily female (12 [80%]) and white (14 [93%]), with one African American. Mean age was 53.1 years (range, 42–63 years). YAs had a mean of 3.3 siblings. YAs' parents ranged n age from 68 to 99 years (mean: 82.6 years). Two YAs were high school graduates; six had at least some college, and seven had attended graduate school. We did not collect education information about the YAs' parents. YAs rated their parents' health at 2.87; none rated their parents' health as poor.

OAs rated their mean depth of EOLPP discussion with their adult children as 4.21; YAs rated their mean depth of discussion with their parents as 4.73, a nonsignificant difference. Eleven OAs said they wanted no heroic measures to prolong their lives, three said maybe or unsure, and one definitely wanted life-prolonging efforts. Nine YAs believed their parents would not want heroic measures, two believed they would, and four did not know. Three OAs and two YAs were familiar with *Five Wishes*.

From the differences that emerged about family discussions, we conceptually organized the responses (Figure) as follows:

- Yes/Yes (n = 9 OAs; n = 10 YAs): Parents are able to share their EOLPP with their children.
- Yes/Not Yet (n = 2 OAs; n = 2 YAs): Parents wish to discuss EOLPP (Yes), but their adult children do not (Not Yet).
- Not Yet/Yes (n = 0 OAs; n = 1 YA): Parents do not talk about EOLPP (Not Yet), but their adult children are willing to hear their wishes (Yes).
- Not Yet/Not Yet (n = 4 OAs; n = 2 YAs): Parents have not discussed EOLPP, and their adult children have not pursued the subject.

**Figure:** Likelihood of planning matrix: conceptual organization of responses from interviewed elders and adult children about whether they discuss end-of-life preparation and preferences.

**ELDERS: Willing to Discuss?**			
*Yes*		*Not Yet*	
Elders talk	Elders try to talk	Elders unwilling or postponing	Elders unwilling or postponing

Planning occurs—information is exchanged	Small exchange of information possible	Small exchange of information possible but unlikely	No planning

Children listen, are receptive	Children cut off conversation	Children instigate discussion; receptive	Children do not instigate discussion
*Yes*	*Not Yet*	*Yes*	*Not Yet*
**ADULT CHILDREN: Willing to Discuss?**			

Eleven OAs reported being comfortable planning ahead and sharing their thoughts about EOLPP (Appendix A, no. 1). Nine OAs already had talked at length with at least one adult child. However, even OAs and their children who discussed EOLPP had not always addressed all issues (Appendix A, no. 2). Six OAs reported trying to talk with their children but being rebuffed or having their children refuse to discuss the OAs' EOLPP (Appendix A, nos. 3–6).

Ten YAs reported talking with their parents about EOLPP (Appendix B). Five YAs either were not yet ready to discuss EOLPP or their parents had not broached the subject with them (Appendix C).

Four OAs and four YAs indicated their openness to discussing EOLPP or their recognition of it as a topic they should discuss but continued to postpone discussing (Appendices D and E). Obstacles to discussing EOLPP fell into five categories:

- **Protection of the children**. OAs believed they needed to shield their adult children from the fact of their parents' death. YAs believed their parents were shielding them.
- **Trust in others to make the decisions**. OAs expressed trust in the family, God, and the physician. YAs mentioned their parents trusted them (children) and God to make end-of-life preparations but did not mention their parents' trust in the physician.
- **Preferences unknown.** OAs expressed not knowing their preferences. No YAs mentioned this as an issue with their own parents, but some did not know their parents' preferences.
- **Family rarely together.** Both OAs and YAs expressed as an impediment to discussing the parents' EOLPP the difficulty of gathering the family and finding an appropriate time to discuss the topic. YAs were more likely than OAs to mention distance and infrequent family visits as obstacles.
- **Fear of death.** OAs expressed fear or a wish to avoid discussing death. One YA indicated her father feared death.

Four facilitators helped OAs talk with their families about EOLPP (Appendix F):

- Acceptance of death,
- Religious faith or spirituality,
- Prior experience with death (especially in regard to life-prolonging measures), and
- Perception of EOLPP discussion as a way to help the family.

Respondents who reported productive EOLPP discussions identified some successful strategies (Appendix G), as follows:

- **A casual approach.** At least four OAs reported mentioning their EOLPP casually to at least one or two children at a time and on an ongoing basis as a primary strategy for discussing the topic. Although not identified as such, YAs' descriptions also sometimes indicated a casual approach (Appendix B).
- **Discussion with one child.** Both OAs and YAs reported differences among children's willingness to discuss EOLPP. Willingness to listen by at least one adult child with whom the elder could comfortably talk helped the elder express EOLPP. Three OAs indicated daughters were easier than sons to talk with about EOLPP, but seven OAs could discuss EOLPP with their sons or found no difference between their sons and daughters. YAs reported observing differences in their siblings' abilities to discuss EOLPP with their parents.
- **Written EOLPP** (Table). Six OAs reported having spoken with their families about an advance directive but had not written their EOLPP. Seven OAs had written, signed, and shared their powers of attorney with their children; four had signed and shared their advance directives. Eight YAs reported their parents had written, signed, and shared their powers of attorney; eight reported their parents had signed and shared advance directives. Additionally, some OAs had given their children detailed instructions about their after-death arrangements.

## Discussion

America has a death-denying culture (13), and people who cannot face death are not likely to be able to discuss EOLPP. The need for education and communication is evident (6,14-18). Only 18% of Americans have living wills (6). People sometimes trust, even prefer, others to make end-of-life decisions for them (16). Although 95% of elders in one study (19) said they "trusted" someone — more often children than spouses — to make decisions for them, fewer than half actually had spoken with the person they expected to make the decision. However, research suggests that discussing EOLPP lightens a family's decision-making burden (20).

Surrogate decisions are problematic (16). In one study, surrogates' decisions were wrong 30% of the time (5), erring mostly toward over-treatment. In reflecting on the hospital as the primary site of death for elders and on the fact that only 22% allow time to plan for death, Kaufman (20) noted, "It is ironic that, in the hospital setting, families are the players with the least knowledge . . . yet they are burdened with what seems to them untenable responsibility." Kaufman observes many families believe they must make life-or-death decisions and "the fact that patients rarely articulate to family or physicians their desires either for life prolongation by technological means or for the cessation of treatment" (p. 36) is a primary difficulty in determining appropriate treatment.

Thus, understanding the process of family EOLPP discussions is important. Our findings contribute to this understanding but are subject to limitations. First, study participants have not yet provided feedback about the validity of our findings (11,21). Second, our participants might differ from the general public in their willingness to discuss EOLPP, as evidenced by their consenting to an interview. Our small sample presumably would not include people who fear death to the extent they would not consent to an interview. Thus our matrix (Figure) assumes that, given the right time and right intervention, all elders and their adult children eventually will discuss EOLPP. However, further research is needed to determine whether an additional category, in fact, exists: a parent/child dyad that might never discuss EOLPP. A revised matrix would need to include this group. Finally, because our sample was primarily female and white, our results might not be generalizable to men or to people of other races/ethnicities; we are targeting additional interviews to men and African Americans. Our recruitment of study participants from the local community council on aging counterbalanced any limitations inherent in the snowball selection technique.

In our study, a casual approach to EOLPP and writing down EOLPP were reported as effective. Both options overcome the obstacles of talking with one child at a time — which potentially could result in family conflicts about the parents' actual EOLPP — and the difficulties of gathering the family at one time and place. Writing EOLPP in some form and giving them to all their children ensures all family members will receive the same message. Even if the children do not read the information when it is written, they will have the parents' preferences when they need them.

Study participants showed interest in learning more about EOLPP. Health care and public health professionals could design interventions targeted toward people in each category of the matrix that would facilitate discussions about EOLPP. Another strategy to facilitate EOLPP discussions is to offer educational sessions that would, for example, explain *Five Wishes*, perhaps even as parent/adult child events, to encourage the dyads to address advance planning. Furthermore, the act of engaging in this interview seemed to spur some participants to begin thinking about their need to address EOLPP; a follow-up of our sample would reveal whether they later discussed EOLPP with their families after participating in our study.

As the older population has increased in the United States, the way elders die has become a public health issue. Our pilot study sheds light on the little-understood process by which elders do or do not discuss their EOLPP with their children. Despite its limitations, the study provided valuable insights from the perspectives of OAs regarding individual barriers and facilitators to discussing the topic. Future research is needed to identify interventions at the interpersonal and societal levels.

## Figures and Tables

**Table:** Actions Regarding Advance Directives Reported by Older Adults (N = 15) and by Younger Adults (N = 15) about Their Parents, Study on End-of-Life Preparation and Preferences, May 2006

Action	Shared and Signed		Discussed, Not Written		No Action/Don't Know	
	OA	YA	OA	YA	OA	YA
Advance directive document	4	8	6	1	5	6
Health care power of attorney/Health care decision maker	7	8	4	3	4	4

OA indicates older adults (aged ≥70 years); YA, younger adults (aged 42–63 years).

## Appendices

### Appendix A. Selected Comments From Older Adults (OAs) Who Had Discussed Their End-of-Life Preparation and Preferences With Their Adult Children, Regardless of Whether the Children Wanted to Discuss the Topic

1. One OA rated her discussion a 7, saying, "My kids know me. They know what I like and what I don't like."
2. Another OA rated her discussion a 3 "because there's lots I would still like to communicate with [my son] about."
3. One OA said, "I gave my daughter an envelope with directives, etc., and she said, 'I don't want to talk about this.' Every time I try to broach the subject, she doesn't want to talk about it. I said, 'Well, when will you talk about it?' She said, 'Well, I am gonna wait until something awful happens.' The envelope is still sealed in her desk."
4. The interviewer asked, "Have you had discussions with your son about what your wishes would be?" The OA replied, "To a degree, but he doesn't wanna talk about it. I am hoping to talk more. I kept some of [my husband's] ashes, and I said to my son one time, 'Well, honey, when I am gone, sprinkle daddy's ashes on top of mine and give it a little shake,' and he said, 'Mother!'"
5. One OA said, "I say anything to them about it and they want me to stop talking about it: 'Don't be talking about it, I don't want to hear it.'"
6. One OA told the interviewer, "I said I don't want to be kept alive . . . but I don't remember which one I told that. It was one of my sons. He just turned it off, so we will see. . . ."

### Appendix B. Selected Comments From Younger Adults Who Had Discussed End-of-Life Preparation and Preferences With Their Parents

1. "[My mother] doesn't like to discuss it head on; she takes a bit of an angle to get to it, but the content was there."
2. "We have talked about it, but we really haven't written anything down. We just have it mentally."
3. "We are starting to talk about what their services should be like, and Mother periodically drops the stuff on me, like, 'Boy, that's always been one of your dad's favorites.' So I write the stuff down real quick."

### Appendix C. Selected Comments From Younger Adults Who Had Not Discussed End-of-Life Preparation and Preferences With Their Parents

1. "They never really said much . . . they did have a living will, but they did that without any real discussions with the rest of us . . . so nobody knew where they were or what they said."
2. "My dad discussed a lot about insurance. . . . But as far as making decisions about end of life, 'if I'm in the hospital, do you disconnect?' or whatever, none of that."
3. "[In] my husband's family . . . there was zero [discussion]. There was nothing."
4. "You don't want to go there. You don't want to approach that in a conversation."

### Appendix D. Selected Comments of Interviewed Older Adults Who Postponed Discussing End-of-Life Preparation and Preferences With Their Adult Children

**General comments**

1. "I realize I gotta do that. That's one thing I keep putting off."
2. "No action. I haven't really wanted to talk about it, but I know I need to."
3. "I haven't taken action, and every time I try, I haven't spoken to my son, but I have a feeling he'll say, 'Oh Mom, let's not worry about that.'"

**Barriers to discussing end-of-life preparation and preferences**

***Protection of the children***

1. "[My children] don't comprehend anything bad pertaining to me or their dad, but sometimes you have to face it. . . . As far as they are concerned, it's 20 years down the road or more, but it's not, but they think it is."
2. "I don't talk to my children about me dying because they are so protective of me. It would hurt them, and I know they don't want to hear anything like that. . . . They don't even want to think about it, so I don't bring up the subject. . . . "

***Trust in family, God, or the physician to make the decisions for them***

1. "I am not saying that when this happens do this, and when this happens do this. I trust my kids; they will make the right decision."
2. "I don't want to be kept alive. If God wants me to go, let Him let me go."
3. "I am gonna tell my doctor that I want a living will and let him put it in his file, and the only way that I want to be put on life support is for him to make decisions that I would come back to some kind of a good way of living."

***Not knowing their preferences***

"That part I haven't said too much. I want them to put me on life support sometimes I think, and then other times I don't."

***Family rarely together***

"I haven't talked to my children about it. I keep saying I am going to, but it's hard to get both of them here at the same time. They are in and out, and about the only time we get together is where we have a lunch or dinner or Christmas, Thanksgiving, or something."

***Fear of death***

1. "I haven't completely got over that fear. . . . Every once in a while, my medications get to a certain point and it seems like I have a different feeling inside, and I think well, you are just gonna die. Now this don't happen often, and then I begin to think, am I ready? So, I still have a little bit, I haven't got to that point yet where it's completely gone. It might not ever be gone on this earth."
2. "I think I live in a little bit of a dream world in that I really avoid unpleasant or sad things."

### Appendix E. Selected Comments From Younger Adults Who Will Not Discuss End-of-Life Preparation and Preferences With Their Parents Who Want to Discuss the Topic

1. "Last Christmas, my mother brought out casket information . . . she was very serious about it . . . she was trying to show us what she had, and my siblings started joking with her. I was afraid she was going to get really upset because she was serious . . . but my siblings didn't want to talk about it, you could obviously tell that they didn't want her thinking and talking about that."
2. "I've actually talked with my siblings about it when the Terri Schiavo case came about, and I had the forms, and I wanted to bring them out too, but the only time we're all together is at the holiday time, so it just sort of seemed like it was a weird time."

### Appendix F. Selected Comments From Interviewed Older Adults About Four Facilitators to Discussing End-of-Life Preparation and Preferences

**Acceptance of death as a part of life**

1. "It does not scare me in any way to talk about death because it's just something that if we live long enough we are going to die, but we don't want to. . . . Sometimes if you are lucky you get to where you can feel more at peace about it."
2. "I was in denial. I am not in denial anymore, having gone through two in the last year. I do not have a negative feeling about death."
3. "I am not morbid by any means and God and Heaven knows I would rather be here. I don't wanna die, but the whole thing about it is, one of these days we're all going to, and why not make the preparations?"
4. "I think when you are dead, you are dead. And I think if you don't have nice memories, that would be terrible, but I am not a religious person anymore."

**Religious faith or spirituality**

1. "When you have faith yourself, it don't supposed to be upsetting to you because we are all born to die, and if you die with Christ, you are gonna live again. So I never think about dying . . . but I hope I be ready when God calls me. I have never seen anybody that still wanted to stay here when time come to go."
2. "You are supposed to be ready and to prepare yourself for this and that's the way I look at it, but it's not easy, unless you are a Christian and you live right, you know there's no problem, you are just as peaceful as that next person."
3. "I am a strong believer in Christ. . . . Death is not with me a sad situation. It's joy. When you see people suffer, you know they are better off gone than to keep suffering."

**Past experience with deaths of friends and family**

"I don't want all of that poked down my nose because you know when I had this surgery, they put all them things in my nose. . . . I really, if it would do any good, I would say it, but with my husband, I saw that [it] didn't do any good."

**A way to help the family**

1. "I have got to help my family. I have got to make some kind of decision when that time comes. I told him I didn't want to be kept alive if I had cancer or something. . . . I've just gotta do it. I need to do it for their sake."
2. "I know that one of these days I am gonna die, and I sure don't wanna be laying there knowing that I am gonna be dying, and them worrying about this, that, and the other."
3. "My son said, 'I am sure glad you made that decision [to have his father cremated] because I would have had a lot of trouble making that decision.' I said, 'Well, Daddy and I talked about it a lot, and we were of the same opinion.'"

### Appendix G Selected Comments From Interviewed Older Adults and Younger Adults About Successful Strategies for Discussing End-of-Life Preparation and Preferences (EOLPP)

**Taking a casual approach**

1. "All along I have mentioned it all the time. It's not just a sit down decision . . . and they have taken it in."
2. "It's just kind of casually. . . . It's casual, but I think they all know exactly what I want."
3. "It's been very casual . . . the fact that we want cremation and no heroics . . . very casual . . . It's never been, 'Let's sit down and talk about this.'"

**Discussing EOLPP with at least one child**

1. "One of my sons would be a one because it makes him nervous, but then the other one that I put in charge of my affairs, I could tell him anything."
2. "My older sister and my brother are open to talking about it. My youngest sister is really not willing to discuss it very much."

**Writing it down**

1. "He had . . . everything printed out, written out. . . . He had already set aside, you know, the power of attorney for the living will and all of that had been done."
2. "She just wrote it and gave it to me. 'Read this and if you have any questions, let me know because this is what you are doing.' I know what she would want. Like if there was some sort of crisis, I wouldn't be wringing my hands wondering, what should I do? I mean, I know exactly."
